# Integrating customer-based brand equity and the theory of planned behavior to predict electric vehicle adoption in China: The moderating role of perceived price

**DOI:** 10.1371/journal.pone.0329224

**Published:** 2025-07-24

**Authors:** Chuan Zheng, Danling Sun, Munirah Khamarudin, Azanin Ahmad, Han Wei, Jie Xu

**Affiliations:** 1 Luzhou Vocational and Technical College, Luzhou, Sichuan, China; 2 School of Business Management, Universiti Utara Malaysia, Sintok, Kedah, Malaysia; 3 Sichuan Vocational College of Chemical Technology, Luzhou, Sichuan, China; 4 School of Literature and Media, Chaohu University, Hefei, China; Xuzhou University of Technology, CHINA

## Abstract

Despite burgeoning global sustainability efforts, the widespread adoption of electric vehicles (EVs) in China faces persistent hurdles, highlighting a critical need to unravel the intricate psychological and brand-related factors that drive consumer purchase decisions. This pioneering study addresses this gap by innovatively integrating customer-based brand equity (CBBE) with the robust theory of planned behavior (TPB), offering a comprehensive framework to predict EV adoption. Drawing on responses from 379 potential EV consumers across six major cities in China, this research employs Partial Least Squares Structural Equation Modeling (PLS-SEM) via SmartPLS 4.0. A two-stage analytical strategy is adopted—validating the measurement model, followed by a rigorous assessment of structural paths including direct, mediating, and moderating relationships among key constructs. Results reveal that all four CBBE components—brand awareness, brand association, perceived quality, and brand loyalty—significantly influence attitude, confirming their critical role in shaping consumer perceptions. The findings demonstrate that attitude, perceived behavioral control, brand association, perceived quality, and brand loyalty significantly influence purchase intention, while subjective norms and brand awareness show no direct effect. Mediation analysis confirms that attitude partially mediates the effects of brand association, perceived quality, and brand loyalty on purchase intention, but not brand awareness. Furthermore, perceived price negatively moderates the relationship between attitude and purchase intention. This research offers groundbreaking insights into brand equity and consumer psychology’s role in accelerating EV adoption. This model provides unprecedented theoretical contributions by linking CBBE and TPB for sustainable consumption. For practitioners, it delivers actionable, evidence-based strategies enabling automakers and policymakers to significantly boost EV market penetration, forging a more sustainable future. This work is a powerful roadmap for unlocking the EV market’s full potential in China and globally.

## Introduction

Transportation-related air pollution represents a critical global challenge, threatening environmental sustainability and public health. Vehicle emissions, particularly fine particulate matter (PM2.5), exacerbate human diseases, with only seven countries meeting the World Health Organization’s (WHO) recommended PM2.5 limit of 5 µg/m³ in 2024 [1]. This underscores the urgency of transitioning to sustainable transportation solutions, as emphasized by Sustainable Development Goal #7 (SDG 7), which advocates for cleaner energy [2]. Electric vehicles (EVs) have emerged as a cornerstone of this transition, with projections indicating that 80–90% of new vehicle sales must be zero-emission by 2050 to meet climate targets [3]. Despite China’s dominance in EV manufacturing, recent trends indicate a slowdown in market growth and a wave of bankruptcies among EV brands, driven by economic uncertainties, the withdrawal of government subsidies, and escalating price wars [4].

These dynamics underscore the critical need to understand consumer decision-making in EV adoption, particularly within China’s competitive and rapidly evolving market. This unique landscape challenges traditional theories of customer-based brand equity (CBBE) [5], which have primarily been applied to established automotive brands with long-standing consumer relationships [6]. Beyond factors like charging infrastructure availability [7], branding has long been recognized as a key determinant of consumer trust and willingness to pay a premium [8–10]. However, its role in shaping consumer attitudes and behavioral intentions in emerging mobility technologies remains underexplored.

The psychological dimensions of branding, particularly the influence on perceived risk, attitude (ATT) formation, and purchase intention (PI), are relevant in the context of EVs. Unlike traditional vehicles, which benefit from deeply ingrained consumer loyalty and habitual decision-making, emerging mobility solutions require consumers to navigate uncertainty, trust deficits, and shifting social norms. Research suggests that consumer adoption of innovative technologies is often moderated by psychological barriers, including perceived control, risk aversion, and normative influences [11–13]. However, how brand-related factors interact with these psychological constructs to shape adoption intentions remains insufficiently studied.

China’s EV markets offer an ideal context to examine these psychological mechanisms, as many nascent domestic brands have rapidly gained prominence despite the absence of long-term brand equity and entrenched consumer loyalty. Unlike legacy automotive brands that rely on historical brand strength, these emerging brands must construct trust and credibility in real-time, often leveraging technological innovation and sustainability narratives as psychological persuasion tools [4]. Moreover, the post-subsidy landscape, regional adoption disparities, and evolving consumer risk perceptions further complicate the branding equation [14]. Understanding the cognitive and affective mechanisms that drive consumer trust and willingness to adopt these technologies is therefore critical.

Building on this premise, the present study develops an integrative theoretical model that synthesizes CBBE with the theory of planned behavior (TPB) [15]. This framework allows us to systematically examine:

i. The direct effects of ATT, perceived behavior control (PBC), and subjective norms (SN) on PI.
ii. The direct effects of CBBE components, e.g., brand awareness (BAW), brand association (BAS), perceived quality (PQ), and brand loyalty (BL), on consumer ATT and PI.
iii. The mediating role of ATT in the relationship between CBBE components and PI.
iv. The moderating influence of perceived price (PP) on the attitude-intention pathway.

This research contributes to the psychological literature on consumer decision-making in three key ways. First, it extends CBBE theory by examining how branding functions as a psychological heuristic in high-risk, technology-driven markets, where traditional branding mechanisms may be less relevant. Second, it enhances understanding of sustainable consumption behaviors by demonstrating how brand-related cognitive and affective processes interact with economic motivations in shaping EV adoption. Third, it offers an empirically validated framework that provides actionable insights for marketers, policymakers, and industry stakeholders, bridging branding theory with behavioral intention models to advance the discourse on consumer psychology in emerging mobility ecosystems.

To address the research questions and achieve its objectives, this study systematically reviews relevant theoretical foundations, integrating the TPB and CBBE frameworks to formulate hypotheses. A detailed methodology then precedes an empirical analysis, which includes descriptive statistics, measurement validation, and structural modeling—encompassing direct, mediation, and moderation effects. The findings are subsequently discussed in relation to the research objectives, culminating in articulated theoretical and managerial implications, along with identified limitations and directions for future research.

## Literature review and hypothesis development

### Theories and framework integration

Recent scholarship increasingly advocates for integrative theoretical models that extend beyond the limitations of traditional behavioral theories. TPB, developed by Ajzen (1991) as an evolution of the Theory of Reasoned Action (TRA) [16], has received extensive empirical validation in explaining PI [17]. However, critiques have emerged regarding TPB’s limited variable scope, particularly its insufficient incorporation of affective and emotional components [18,19]. This gap weakens its explanatory power in complex, emotionally charged decisions such as EV adoption [20]. To address this, integrating CBBE provides a valuable theoretical extension [21]. CBBE encompasses four core dimensions—BAW, BAS, PQ, and BL—each contributing to consumer decision-making through enhanced recognition, emotional engagement, and perceived value. Incorporating CBBE into the TPB framework enhances its ability to predict PI in markets characterized by innovation and uncertainty [22].

Social Identity Theory (SIT), developed by Tajfel (1986) [23], which is originally refined from Tajfel and Turner (1979) [24], offers complementary theoretical insights that substantially enrich discourse. SIT posits that individuals’ self-conceptualization is partially constructed through their group affiliations, which subsequently manifests in distinctive attitudinal and behavioral patterns. Within brand-related contexts, this theoretical framework elucidates how consumers who identify with specific brands develop preferential attitudes and behavioral dispositions toward these entities [25]. The theory has demonstrated empirical robustness in explicating consumer behavior toward specific product categories, notably EVs [26]. SIT effectively illuminates the psychological mechanisms through which individuals exhibit preferential treatment toward in-groups, manifesting in heightened brand loyalty and preference for high-equity brands [27].

Integrating CBBE with TPB, bridged by SIT, yields a more comprehensive framework for understanding consumer behavior. This synthesis is particularly salient in the EV context, where sociocultural identity and brand perceptions jointly shape adoption behavior. Although extensions of TPB have included factors such as EV experience [28], moral norms [29], and environmental or innovation orientations [30], the model still struggles to capture deeper sociocultural variables— including personal values, normative pressures, and belief systems—that substantially influence behavioral outcomes [31–33].

### TPB determinants in EV adoption

This systematic integration of theoretical frameworks illuminates several critical hypotheses regarding consumer behavior toward EVs. TPB posits three fundamental determinants of behavioral intentions: ATT, SN, and PBC [15]. ATT, conceptualized as an individual’s evaluative disposition toward performing a specific behavior, is fundamentally shaped by underlying behavioral beliefs [15]. The theoretical framework suggests that more favorable attitudinal dispositions engender stronger behavioral intentions. Similarly, SN, influenced by normative beliefs and motivational factors to comply with social pressures, demonstrates a positive association with behavioral intentions [15]. PBC refers to the extent to which an individual perceives the ease or difficulty of performing a specific behavior, with higher perceived control associated with a stronger intention to engage in that behavior [15]. Empirical evidence consistently establishes ATT as a pivotal predictor of PI across diverse consumption domains, including energy-efficient appliances [34], fashion products [35], M-health applications [36], green products [37], personal care items [38], and fast-food consumption [39]. SN has also been consistently identified as a critical determinant of PI, particularly for green product adoption [40–42]. Complementing these, PBC is robustly validated for its significant and positive impact on PI across various contexts [43,44]. Therefore:

**H1** ATT demonstrates a positive association with consumer PI.

**H2** SN exhibits a positive relationship with consumer PI.

**H3** PBC manifests a positive influence on consumer PI.

### CBBE components in EV adoption

The CBBE framework, originally conceptualized by Keller (1993) [5], emphasizes the pivotal role of BAW and BAS in shaping consumer responses [45,46]. This theoretical paradigm, influenced by consumer knowledge structures and marketing initiatives, has demonstrated substantial empirical relevance in brand development processes [47,48]. The seminal Aaker (1991) [49] model established a foundational framework for understanding brand equity dimensions [50,51]. However, the dynamic evolution of consumer behavior necessitates continuous reassessment of CBBE dimensions, particularly within the emergent EV market context. To address these theoretical gaps, this investigation employs BAW, BAS, PQ, and BL as fundamental CBBE dimensions to examine brand equity’s influence on consumer responses, conceptualizing ATT and PI as outcome variables, with PP as a moderating construct.

BAW, operationalized as consumers’ capacity to recognize and recall a brand with sufficient precision to facilitate purchase decisions [52], encompasses brand recognition, recall, and general awareness dimensions [53]. This construct constitutes the foundational element of brand equity upon which subsequent brand-related cognitive structures are constructed [54]. Although frequently underestimated in empirical investigations, BAW demonstrates substantial influence on perceptual processes, brand selection mechanisms, and loyalty formation [54]. The construct exhibits predictive validity regarding attitudinal formations through enhanced information accessibility and familiarity mechanisms, culminating in favorable brand evaluations [55,56].

Through the theoretical lens of SIT, this heightened familiarity enhances individuals’ self-conceptualization through alignment with their social identity constructs. Consequently, BAW signifies brand commitment and motivates consumer consideration during purchase deliberations, fostering positive attitudinal dispositions and elevated PI [57]. The construct demonstrates particular salience in shaping brand attitudes, influencing evaluative processes, emotional responses, and behavioral propensities [58].

Empirical evidence substantiates BAW’s enhancement of core-brand ATT, PI, and repurchase behaviors [59,60]. However, the magnitude of its influence demonstrates variability across empirical investigations, with select studies indicating minimal impact [61,62]. Consumer BAW has been identified as a significant determinant in shaping PI [63], with numerous studies documenting positive associations between BAW and PI [64,65]. This empirical heterogeneity necessitates context-specific analysis to elucidate BAW’s true effect on attitudinal formation. Therefore:

**H4a** BAW demonstrates a positive association with consumer PI.

**H5a** BAW exhibits a positive relationship with consumer ATT.

BAS, conceptualized by Aaker (1991) [49] as multidimensional memory linkages between brands and consumer cognitions, are shaped through diverse experiential and exposure mechanisms [66]. These associative networks constitute the fundamental essence of brand identity, facilitating the activation of brand-related cognitive structures and establishing comprehensive mental representations [67]. The construct encompasses specific attributes, benefits, and characteristic dimensions linked to brands within consumers’ cognitive frameworks. CBBE, operationalized as the value enhancement a brand designation confers upon products or services [49], leverages these associative networks to construct positive brand imagery.

Drawing on SIT, individuals form significant aspects of their identity through brand affiliations. Positive BAS reinforce consumers’ social identity and self-concept, fostering favorable brand attitudes. Moreover, congruent BAS demonstrate influence through peripheral persuasion routes, generating enhanced positive brand attitudes [68]. Attitudinal dispositions toward brands, conceptualized as holistic evaluations of brand-related associative networks in memory structures, are substantially influenced by positive brand cognitions and thought processes [69,70]. Furthermore, BAS exhibits positive influence on consumer behavioral intentions across various contexts, including mobile data service utilization [71], with consistent empirical documentation of positive associations between BAS and PI [72]. Therefore:

**H4b** BAS has a positive association with PI.

**H5b** BAS has a positive association with ATT.

PQ, operationalized as consumers’ subjective judgment regarding a product’s holistic superiority [73], demonstrates conceptual distinction from objective quality metrics [74]. The construct encompasses consumers’ evaluative assessment of product excellence relative to intended functionality [75] and represents a distinct attitudinal formation from objective quality measurements [76]. Furthermore, seminal theoretical frameworks emphasize PQ’s enhancement of consumers’ social identity constructs and self-conceptualization processes [49,77]. SIT posits that individuals demonstrate preference for brands that reflect desired identity constructs, with elevated PQ reinforcing positive self-image formation and fostering favorable brand attitudes [78]. PQ, conceptualized as a consumer attitudinal formation derived from expectation-performance comparisons [79], demonstrates positive associations with attitudes toward private label products [80] and correlates with positive brand attitudinal formations [78]. Moreover, store brands’ PQ demonstrates significant influence on consumer attitudinal formations and purchase behaviors [81]. Additionally, robust empirical evidence substantiates positive associations between PQ and behavioral intentions [82–85]. Therefore:

**H4c** PQ demonstrates a positive association with PI.

**H5c** PQ exhibits a positive relationship with ATT.

BL, conceptualized as repetitive purchase behavior toward specific brands coupled with emotional preference formations [86], encompasses both attitudinal and behavioral loyalty dimensions [87]. The robustness of associations between positive brand attitudes and advocacy behaviors is emphasized [88]. Despite its attitudinal component [89], affective loyalty, which engenders robust emotional bonds and positive brand attitudes, demonstrates particularly predictive validity regarding consumer behavior [90]. This emotional attachment mechanism aligns with SIT proposition that individuals derive significant identity components from preferred brand affiliations, enhancing self-conceptualization processes. Consequently, BL engenders consistent preferential dispositions and behavioral intentions toward specific brands, demonstrating substantial influence on attitudes toward co-branded entities [9]. It is further posited that BL encompasses consistent purchase patterns regarding specific goods or services, maintained despite external influential factors [90]. Extensive empirical investigations have examined BL’s impact on PI across various contexts [91–96]. Therefore:

**H4d** BL demonstrates a positive association with PI.

**H5d** BL exhibits a positive relationship with ATT.

### Mediation of ATT

Within the TPB framework, ATT, SN, and PBC function as mediating mechanisms between background factors and behavioral intentions [97]. Robust empirical evidence substantiates the critical influence of specific attitudinal formations on consumer decision processes [98]. Although extant literature implicitly acknowledges ATT’s mediating function [99–101], significant theoretical gaps persist regarding its impact within the CBBE-consumer response paradigm. Empirical evidence substantially supports ATT’s mediating role among trust, SN, PBC, and PI [102,103]. Similarly, research underscores ATT’s critical mediating function between brand knowledge, religiosity, customer experience, consumer-cause identification, and subsequent behavioral outcomes including PI and loyalty formations [104–106]. These findings receive further empirical corroboration through multiple investigations [107,108], highlighting ATT’s mediating function between various predictor variables and PI. Nevertheless, the specific mediating mechanism of ATT between CBBE dimensions and consumer responses remains inadequately investigated [109]. Therefore:

**H6a** ATT mediates the relationship between BAW and PI.

**H6b** ATT mediates the association between BAS and PI.

**H6c** ATT mediates the relationship between PQ and PI.

**H6d** ATT mediates the association between BL and PI.

### Moderation of PP

PP, characterized as consumers’ subjective price perceptions at specific temporal points [110], consistently demonstrates a crucial influence on consumer decision processes [111,112]. The construct encompasses consumers’ subjective interpretations of objective price metrics [113], demonstrating significant influence on customer satisfaction, loyalty formations, and PI [114,115]. PP demonstrates robust associations with perceived value constructs, with both quality perceptions and price interpretations contributing to customer satisfaction formations [116,117]. Despite its theoretical significance, PP’s moderating influence on ATT-PI relationships remain inadequately explored. Empirical evidence suggests differential responses to attitudinal formations between price-sensitive and price-insensitive consumer segments [118]. Research indicates that perceived value, incorporating price perceptions, moderates the relationships between quality perceptions and PI across diverse product categories [119]. Within the EV context, PP can be conceptualized as consumers’ subjective price perceptions relative to conventional fuel vehicles [120]. Therefore:

**H7** The positive relationship between ATT and PI demonstrate greater magnitude under conditions of lower PP.

**Fig 1:**
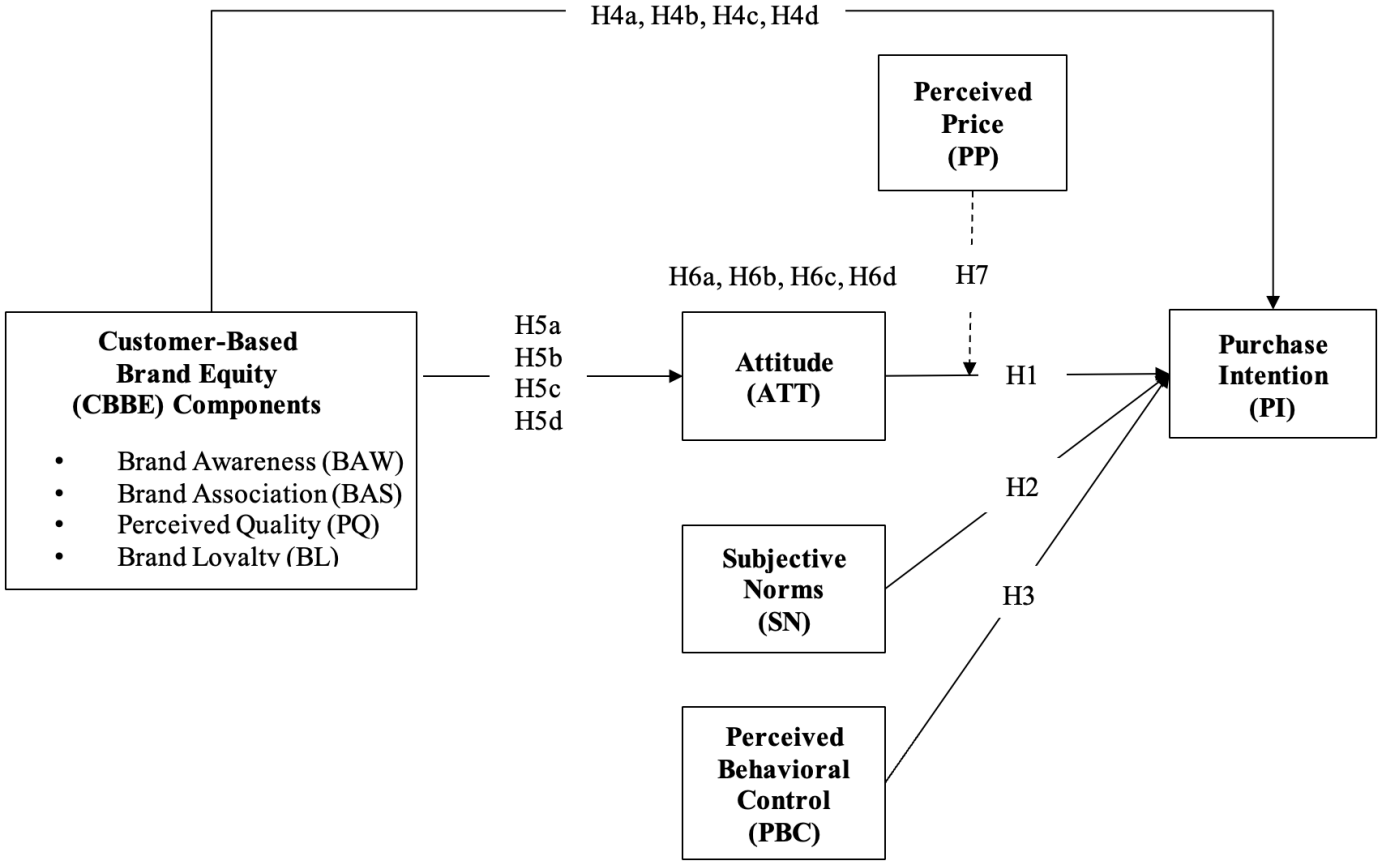
The conceptual research model.

  The proposed conceptual research model is presented in Fig 1.

## Methodology

Given the inherent challenges of accessing a randomized sample within China’s diverse and geographically dispersed EV consumer population, the study’s methodological design favored quota convenience sampling. This approach is specifically chosen to efficiently target and ensure representation across key demographic subgroups, such as urban versus rural consumers and varying income levels, thereby balancing representativeness with practical data collection exigencies. Rather than previous studies focusing only on provinces such as Tianjin [121,122], subnational regions such as Jiangsu, or urban areas such as Shanghai [123], Hefei [124], Beijing, Shanghai, Guangzhou and Shenzhen [125], we aim at China’s top six cities with the highest EV ownership rates (e.g., Shanghai, Beijing, Shenzhen, Guangzhou, Hangzhou, and Chengdu). These cities, chosen for their top six highest NEV numbers in 2023 according to the Ministry of Public Security (MPS) of China, serve as EV demonstration pilot cities, where consumers have greater familiarity with EVs and associated policies. As first- or second-tier cities with elevated per capita GDP and substantial vehicle ownership, they provide a comprehensive and representative view of potential EV buyers, enhancing the generalizability and robustness of the findings.

The questionnaire of the study is organized into two parts, with the first part covering demographic characteristics: age, gender, educational attainment, yearly household income, and city of residence in China. The subsequent part of the questionnaire comprised a series of statements designed to assess all variables. The selection of specific measurement scales for each construct is rigorously guided by principles of theoretical consistency and empirical validity, leveraging instruments extensively validated in prior research. As detailed in Table 1, the items for PI are adopted from a comparable study within the EV adoption context [126]. Measures for ATT, SN, and PBC are primarily derived from established TPB literature [127]. This study also involved careful wording adjustments to explicitly align with the EV context in China, ensuring adherence to the Target, Action, Context, and Time (TACT) principles for TPB constructs [128,129]. Similarly, the CBBE components are operationalized using scales drawn from highly cited works in brand management [130]. Finally, the PP scale is adapted from studies examining consumer perceptions of cost within new technology adoption frameworks [111,120].

**Table 1:** Measurement of constructs.

Variable	Items	Statement	References
PI	PI1	I would consider purchasing an EV.	[126]
	PI2	I intend to try an EV.	
	PI3	I plan on buying an EV.	
	PI4	I am interested in tasting an EV.	
ATT	ATT1	I think buying an EV is a good choice.	[127]
	ATT2	I think it is very necessary to use EVs.	
	ATT3	I think buying an EV is a wise idea.	
	ATT4	I am interested in EVs.	
SN	SN1	Many people who are important to me are considering purchasing an EV.	
	SN2	Many people who are important to me would approve of me purchasing an EV.	
	SN3	Many people who are important to me would want me to purchase an EV.	
PBC	PBC1	Whether or not to purchase an EV is completely decided by me.	
	PBC2	I have complete control over whether or not to purchase an EV.	
	PBC3	I can largely decide whether or not to buy an EV at home.	
BAW	BAW1	I know what X looks like.	[130]
	BAW2	I can recognize X among other competing brands.	
	BAW3	I am aware of X.	
BAS	BAS1	Some characteristics of X come to my mind quickly.	
	BAS2	I can quickly recall the symbol or logo of X.	
	BAS3	I have difficulty in imagining X in my mind. (r)	
PQ	PQ1	X is of high quality.	
	PQ2	The likely quality of X is extremely high.	
	PQ3	The likelihood that X would be functional is very high.	
	PQ4	The likelihood that X is reliable is very high.	
	PQ5	X must be of very good quality.	
	PQ6	X appears to be of very poor quality. (r)	
BL	BLO1	I consider myself to be loyal to X.	
	BLO2	X would be my first choice.	
	BLO3	I will not buy other brands if X is available at the store.	
PP	PP1	EVs are more expensive than traditional fuel vehicles.	[111,120]
	PP2	Buying a traditional fuel car may enjoy a bigger discount than buying an EV.	
	PP3	The maintenance cost of EVs may be higher than that of traditional fuel vehicles.	

Note: PI = Purchase intention, ATT = Attitude, SN = Subjective norms, PBC = Perceived behavioral control, BAW = Brand awareness, BAS = Brand association, PQ = Perceived quality, BL = Brand loyalty, PP = Perceived price.

Furthermore, all statements are assessed applying a five-point scale, where “1” represents “Strongly disagree” and “5” represents “Strongly agree.” The initial questionnaire is in English, and it is interpreted into Chinese by two language experts to ensure linguistic equivalence. The finalized questionnaire was developed based on a pilot test with 50 consumers from the designated six cities, and the sequence of items is randomized to mitigate potential carryover effects.

The survey was exclusively distributed via an online platform named Wenjuanxing (https://www.wjx.cn/), where participants were provided with an informed consent statement prior to commencing the survey. Consent was obtained in written form, as participants have to agree to confirm their willingness to participate after reading the consent information. No minors are involved in the study, and all participants are adults who provide their consent voluntarily. The online survey took place in June and July 2024. This research employed G-power to determine a minimum sample size of 109. Following the guidelines by Nunnally (1978) [131], the survey aimed to secure 400 responses. Finally, valid responses from 379 individuals who are potential EV consumers, filtered by the question “Are you planning to buy an electric car within the next one year?”

## Results

### Descriptive analysis

As shown in Table 2, the sample consists of 54.90% males and 45.10% females. While the selected cities generally have balanced gender ratios, this slight male skew aligns with observed trends indicating higher EV purchase interest and new technology adoption among males. In terms of age, 35.60% of respondents are aged between 26 and 35, followed by 36–45 years (23.5%), 46–55 years (20.1%), 18–25 years (15.8%), and 56–70 years (5.3%). This suggests a younger demographic, which is typically more open to adopting new technologies like EVs. A significant portion of the sample holds higher education degrees: 46.2% with a bachelor’s degree and 38.8% with an associate’s degree. Only 5% have a master’s degree or higher, while 10% have a high school education or less. Most respondents report an annual household income of 100,000–200,000 RMB (33.2%), 200,000–300,000 RMB (36.7%), or 300,000–500,000 RMB (19%). Lower income groups (100,000 RMB or below, 4.7%) and higher income groups (500,000 RMB or above, 6.3%) are less represented. This income distribution indicates that the sample possesses the financial capacity to consider EV purchases, allowing for a nuanced analysis of how PP impacts PI within various income brackets. Respondents from the designated six major Chinese cities with high EV adoption rates: Shanghai (22.16%), Beijing (20.05%), Shenzhen (17.94%), Guangzhou (14.25%), Hangzhou (13.46%), and Chengdu (12.14%).

**Table 2:** Demographic characteristics of the sample.

Variable	Category	Frequency	Percentage
Gender	Male	208	54.90%
	Female	171	45.10%
Age	18-25 years	60	15.80%
	26-35 years	134	35.40%
	36-45 years	89	23.50%
	46-55 years	76	20.10%
	56-70 years	20	5.30%
Educational attainment	High school and below	38	10.00%
	Associate’s degree	147	38.80%
	Bachelor’s degree	175	46.20%
	Master’s degree or above	19	5.00%
Yearly household income	100,000 RMB or below	18	4.70%
	100,000–200,000 RMB	126	33.20%
	200,000–300,000 RMB	139	36.70%
	300,000–500,000 RMB	72	19.00%
	500,000 RMB or above	24	6.30%
City of residence	Shanghai	84	22.16%
	Shenzhen	76	20.05%
	Beijing	68	17.94%
	Guangzhou	54	14.25%
	Hangzhou	51	13.46%
	Chengdu	46	12.14%

### Measurement model

Partial least squares (PLS) modeling is utilized using SmartPLS 4.0 to evaluate both the measurement and structural models [132]. PLS is notably appropriate for this study since it does not necessitate normality in the data distribution, a common issue in survey research [133]. Given the single-source data collection, common method variance (CMV) is assessed by specifically through the examination of full collinearity [134,135]. Variance Inflation Factor (VIF) is computed to evaluate multicollinearity among predictor variables. This approach involves regressing all variables against a common factor, where a VIF value of ≤ 3.3 indicates an absence of substantial bias. The results showed VIF values below 3.3 (see in Table 3), confirming that CMB is not a significant issue in this dataset.

**Table 3:** Full collinearity testing.

PI	ATT	SN	PBC	BAS	BAW	PQ	BL	PP
1.822	1.857	1.532	1.529	1.642	1.689	1.822	1.652	1.348

Note: PI = Purchase intention, ATT = Attitude, SN = Subjective norms, PBC = Perceived behavioral control, BAW = Brand awareness, BAS = Brand association, PQ = Perceived quality, BL = Brand loyalty, PP = Perceived price.

Utilizing methodology by Anderson & Gerbing (1988) [136], this study rigorously evaluated the measurement model to confirm instrument validity and reliability, following established guidelines [137,138]. The measurement model is assessed for validity and reliability using factor loadings, average variance extracted (AVE), and composite reliability (CR) [139]. Criteria for acceptability are loadings ≥ 0.5, AVE ≥ 0.5, and CR ≥ 0.7. Table 4 shows that all AVEs are above 0.5, CRs exceed 0.7, and most loadings are above 0.708, demonstrating convergent validity. Internal consistency is confirmed with Cronbach’s Alpha (CRA) and CR values over 0.7 [140]. Discriminant validity is evaluated by comparing AVE values and their square roots. All AVEs and their square roots exceed 0.5, and each square root is greater than its correlation with other constructs, confirming discriminant validity. Thus, the measurement model is consistent and robust. Table 4 provides the square roots of AVE and their correlations, supporting these findings.

**Table 4:** Measurement model for constructs.

Variables	Items	Factor Loadings	VIF	CRA	CR	AVE
PI	PI1	0.851	2.125	0.879	0.917	0.733
	PI2	0.850	2.147			
	PI3	0.857	2.230			
	PI4	0.867	2.316			
ATT	ATT1	0.857	2.185	0.872	0.913	0.723
	ATT2	0.835	2.040			
	ATT3	0.852	2.149			
	ATT4	0.857	2.246			
SN	SN1	0.840	1.729	0.828	0.897	0.744
	SN2	0.868	2.019			
	SN3	0.879	1.983			
PBC	PBC1	0.858	2.053	0.837	0.902	0.754
	PBC2	0.847	1.800			
	PBC3	0.899	2.109			
BAS	BAS1	0.873	2.054	0.848	0.908	0.767
	BAS2	0.879	2.124			
	BAS3	0.876	1.994			
BAW	BAW1	0.851	1.781	0.826	0.896	0.742
	BAW2	0.857	1.888			
	BAW3	0.876	1.987			
PQ	PQ1	0.843	2.456	0.918	0.936	0.708
	PQ2	0.850	2.581			
	PQ3	0.857	2.589			
	PQ4	0.832	2.359			
	PQ5	0.834	2.354			
	PQ6	0.833	2.412			
BL	BL1	0.861	1.968	0.837	0.902	0.754
	BL2	0.868	1.975			
	BL3	0.876	1.935			
PP	PP1	0.863	1.877	0.802	0.883	0.715
	PP2	0.816	1.699			
	PP3	0.858	1.655			

Note: PI = Purchase intention, ATT = Attitude, SN = Subjective norms, PBC = Perceived behavioral control, BAW = Brand awareness, BAS = Brand association, PQ = Perceived quality, BL = Brand loyalty, PP = Perceived price, VIF = Variance Inflation Factor, CRA = Cronbach’s Alpha, CR = Composite Reliability, AVE = Average Variance Extracted.

In addition to AVE, we employed the Heterotrait-Monotrait (HTMT) criterion for discriminant validity [141,142]. According to these criteria, HTMT values should be ≤ 0.85 for stricter evaluation and ≤ 0.90 for a more lenient assessment. As depicted in Table 5, all HTMT values (upper triangle) are lower than the more rigorous standard of ≤ 0.85, indicating distinctiveness among the nine constructs. Furthermore, the Correlation Coefficients between the latent constructs are also analyzed. As presented in the higher triangle of Table 5, all HTMT values are below the threshold of 0.85, indicating discriminant validity. Diagonal values, representing the square root of AVE, confirm convergent validity as all values exceed the 0.70 threshold. As presented in the lower triangle of Table 5, all diagonal values exceed the recommended threshold of 0.70, confirming convergent validity, along with all correlation coefficients statistically significant at p < 0.05 indicating reliable associations between the constructs. Additionally, we assess the overall model fit. The Standardized Root Mean Square Residual (SRMR) for the estimated model is 0.047, which is below the commonly accepted threshold of 0.08, indicating a good model fit [143]. The Normed Fit Index (NFI) for the estimated model is 0.847, suggesting a reasonable fit, especially in the context of PLS-SEM [144]. The overall assessment of these metrics confirms that our model adequately represents the observed data.

**Table 5:** Correlation Coefficients and Heterotrait-Monotrait (HTMT).

Construct	1	2	3	4	5	6	7	8	9
1.ATT	**0.850**	0.551	0.502	0.568	0.482	0.592	0.603	0.523	0.505
2.BAS	0.475***	**0.876**	0.528	0.542	0.483	0.559	0.565	0.392	0.482
3.BAW	0.427***	0.443***	**0.861**	0.555	0.553	0.571	0.521	0.412	0.549
4.BL	0.487***	0.459***	0.462***	**0.869**	0.468	0.551	0.548	0.381	0.517
5.PBC	0.413***	0.408***	0.463***	0.397***	**0.868**	0.459	0.507	0.242	0.510
6.PQ	0.530***	0.493***	0.497***	0.484***	0.403***	**0.842**	0.581	0.384	0.497
7.PI	0.528***	0.488***	0.444***	0.471***	0.440***	0.523***	**0.856**	0.477	0.494
8.PP	−0.439***	−0.325***	−0.337***	−0.311***	−0.199***	−0.329***	−0.405***	**0.846**	0.357
9.SN	0.431***	0.404***	0.455***	0.433***	0.428***	0.432***	0.422***	−0.291***	**0.862**

Notes: i) Values in the upper triangle are the Heterotrait-Monotrait (HTMT) ratio of correlations; ii) Diagonal values in bold represent the square root of the Average Variance Extracted (AVE); iii) Values in the lower triangle are the Correlation Coefficients with p value of latent variable correlations (***p < 0.001, **p < 0.01, *p < 0.05). iv) PI = Purchase intention, ATT = Attitude, SN = Subjective norms, PBC = Perceived behavioral control, BAW = Brand awareness, BAS = Brand association, PQ = Perceived quality, BL = Brand loyalty, PP = Perceived price.

### Structural model

#### Model assessment.

In line with the recommendations [139,145], the multivariate skewness and kurtosis are assessed. Mardia’s test for multivariate normality is utilized to evaluate skewness and kurtosis in the dataset. Non-significant skewness and kurtosis values indicate that the data meet the multivariate normality assumption [146]. The findings indicated that the data is not multivariate normal, with Mardia’s multivariate skewness (β = 4.408, p < 0.01) and Mardia’s multivariate kurtosis (β = 92.713, p < 0.01). Consequently, path coefficients, standard errors, t-values, and p-values for the structural model are estimated using a bootstrapping procedure with 10,000 samples conducted in SmartPLS 4.0 [147].

A VIF value greater than 5 typically indicates high multicollinearity [148]. It is recommended that an ideal VIF value should be less than 3 to indicate non-problematic multicollinearity [149]. The findings, as shown in Table 4, reveal that all VIF values for the items are below 3. Therefore, it can be inferred that multicollinearity does not present an issue in this study. The reliability of constructs is assessed using Cronbach’s alpha and composite reliability. Factor loadings above 0.7 indicate acceptable indicator reliability [140]. The predictive power of the model is evaluated using R^2^ statistics, which represents the variance explained in each endogenous construct. Furthermore, R^2^ is employed to assess explanatory power and predictive accuracy. Higher R² values signify greater explanatory power, with the range spanning from 0 to 1 [149]. 0.20 or above indicates high explanatory power [150]. In this study, the R² values are 0.389 for PI and 0.472 for ATT, indicating that the model exhibits substantial explanatory power.

#### Direct effect.

In response to critique that p-values alone are inadequate for assessing hypothesis significance, which advocates for using a combination of criteria including p-values, confidence intervals, and effect sizes [151]. Table 6 presents the summary of the criteria used to test the hypotheses developed. First, the direct effects of the predictors on PI are examined. As presented in Table 6, the analysis confirms that several hypotheses achieved statistical significance. Specifically, H1 (ATT - > PI, β = 0.140, p < 0.05), H3 (PBC - > PI, β = 0.113, p < 0.05), H4b (BAS - > PI, β = 0.123, p < 0.05), H4c (PQ - > PI, β = 0.135, p < 0.05), H4d (BL - > PI, β = 0.118, p < 0.05), H5b (BAS - > ATT, β = 0.193, p < 0.001), H5c (PQ - > ATT, β = 0.279, p < 0.001), H5d (BL - > ATT, β = 0.216, p < 0.001), and H7 (PP × ATT - > PI, β = −0.170, p < 0.001) are all statistically significant, as indicated by their respective p-values and confidence intervals, which do not cross zero. These results provide empirical support for these proposed relationships within the model. Conversely, hypotheses H2 (SN - > PI, β = 0.045, p > 0.1) and H4a (BAW - > PI, β = 0.039, p > 0.1) do not meet the threshold for statistical significance, with p-values exceeding 0.05 and confidence intervals encompassing zero, leading to their rejection.

**Table 6:** Hypothesis testing: Direct, mediated, and moderated effects.

Relationship		Std. Beta	Std. Error	t-values	p-values	BCI LL	BCI UL	Conclusion
***Direct Effect***								
H1	ATT - > PI	0.140	0.055	2.520	0.012	0.034	0.252	Supported
H2	SN - > PI	0.045	0.047	0.950	0.342	−0.048	0.138	Not Supported
H3	PBC - > PI	0.113	0.048	2.372	0.018	0.017	0.205	Supported
H4a	BAW - > PI	0.039	0.053	0.741	0.459	−0.063	0.143	Not Supported
H4b	BAS - > PI	0.123	0.048	2.547	0.011	0.027	0.215	Supported
H4c	PQ - > PI	0.135	0.054	2.494	0.013	0.029	0.245	Supported
H4d	BL - > PI	0.118	0.050	2.371	0.018	0.023	0.218	Supported
H5a	BAW - > ATT	0.103	0.051	1.998	0.046	−0.002	0.201	Supported
H5b	BAS - > ATT	0.193	0.048	4.002	0.000	0.099	0.287	Supported
H5c	PQ - > ATT	0.279	0.051	5.445	0.000	0.177	0.377	Supported
H5d	BL - > ATT	0.216	0.052	4.153	0.000	0.116	0.318	Supported
***Mediation Effect***								
H6a	BAW - > ATT - > PI	0.016	0.010	1.644	0.050	0.003	0.035	Not Supported
H6b	BAS - > ATT - > PI	0.040	0.014	2.809	0.002	0.020	0.067	Supported
H6c	PQ - > ATT - > PI	0.035	0.013	2.518	0.006	0.015	0.059	Supported
H6d	BL - > ATT - > PI	0.028	0.012	2.388	0.008	0.012	0.051	Supported
***Moderation Effect***								
H7	PP x ATT - > PI	−0.170	0.046	3.712	0.000	−0.264	−0.084	Supported

Note: i) Note: PI = Purchase intention, ATT = Attitude, SN = Subjective norms, PBC = Perceived behavioral control, BAW = Brand awareness, BAS = Brand association, PQ = Perceived quality, BL = Brand loyalty, PP = Perceived price. ii) Confidence interval with a bootstrapping of 10,000 is used.

#### Mediation effect.

To test mediation effects, bootstrapping is utilized to measure the indirect effect in this study. If the confidence interval does not encompass zero, it can be concluded that significant mediation exists [152,153]. As shown in Table 6, BAS - > ATT - > PI (β = 0.040, p < 0.05), PQ - > ATT - > PI (β = 0.035, p < 0.05), and BL - > ATT - > PI (β = 0.028, p < 0.1) are all significant. Their bias-corrected 95% confidence intervals also do not include zero, corroborating our findings. Thus, H6b, H6c, and H6d are all supported. In contrast, BAW - > ATT - > PI (β = 0.016, p > 0.1) is not significant, and thus H6a is not supported, consistent with the rejection of H5a. Additionally, the Variance Accounted For (VAF) method is used to assess the mediation level with values between 20% and 80% indicating partial mediation, values below 20% suggesting no mediation, and values above 80% indicating full mediation [148]. Based on this, ATT serves as a partial mediator in the effects of BAS, PQ, and BL on PI.

#### Moderation effect.

**Fig 2:**
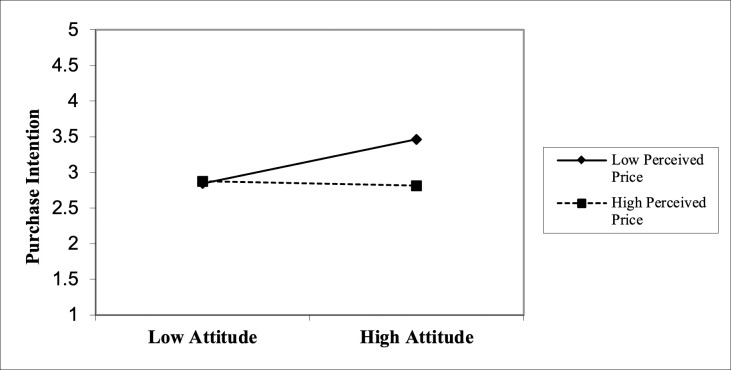
Moderating effect of perceived price on the relationship between attitude and purchase intention.

Table 6 provides a summary of the findings related to the moderating effect of PP. The hypothesis that PP moderates the relationship between ATT and PI is supported by the data. The f² values are defined as measure the impact of the moderator on the endogenous construct, with values of 0.02, 0.15, and 0.35 indicating small, medium, and large effects, correspondingly [154]. The observed moderating effect (f² = 0.064) of PP is thus considered to be of medium strength. Fig 2 illustrates the interplay of PP on the relationship between ATT and PI. The lines represent the effects of the moderator at varying levels. The findings suggest that a low PP is associated with higher levels of both intention and behavior, whereas a higher PP (High PP) is associated with lower levels of PI, while a lower PP (Low PP) is associated with a higher level of PI. This suggests that the relationship between ATT and intention is stronger when the PP is lower. Therefore, the findings corroborate the H7.

## Discussion

Building upon our empirical analysis and grounded in TPB and CBBE frameworks, this discussion synthesizes the key findings that illuminate the factors shaping consumers’ PI for EVs in China. We explore the direct, mediation, and moderation effects identified in our research, thereby addressing the four key research questions and enhancing the understanding of consumer behavior in this rapidly evolving market.

### Direct effect

First, the findings confirm that the three TPB predictors influence consumer PI for EVs. Consistent with previous studies that employed TPB as a theoretical foundation, ATT consistently emerges as a primary determinant in intentions related to EV adoption [155–157]. However, the influence of SN and PBC on PI appears to vary across studies. Some researchers highlight a stronger impact of SN on intention [158,159], whereas others report no significant effect of SN on behavioral intention [159–162], a result that aligns with this study’s findings. Likewise, while ATT is often identified as the strongest predictor of intention [163,164], confirming this study’s outcome, there are instances where ATT or PBC holds a more prominent role. These discrepancies in literature can be attributed to several factors, including variations in the behavior under study, differences in the demographic and cultural contexts of the target populations, the time of the study, and the specific metrics used to operationalize the TPB constructs [15].

Moreover, the study offers support for CBBE’s significant influence on PI of EVs, extending beyond the brand of the EV itself [165]. While prior study indicates a direct and significant effect of BAW is indicated on PI [166], the results of the current study indicate that there is no significant direct effect of BAW on PI. Moreover, the alternative mechanism of BAW effect on PI through the mediation of ATT is also rejected. Therefore, it is concluded that the total effect of BAW on PI is not significant in the context of EVs. These findings may be explained by the unique nature of EV consumer behavior. In high-involvement categories like EVs, which require significant financial investment, consumers may prioritize brand-specific qualities over general BAW. This suggests that attributes such as BAS, PQ and BL, rather than mere awareness, play a more critical role in shaping PI of EVs. Consequently, EV brands should focus on enhancing PQ and building strong associations and reliability to effectively influence consumer decisions in this high-stakes market.

Additionally, the positive and committed relationship of all CBBE elements (BAW, BAS, PL and BL) and ATT is confirmed. It reinforces the multidimensional nature of brand equity, highlighting the interconnectedness of its elements and their collective impact on consumer ATT [59,109,167]. This finding supports the theoretical frameworks that advocate for a holistic approach to brand equity, suggesting that improvements in one dimension can positively influence others. Additionally, it effectively links the TPB with CBBE elements [59,168,169], integrating brand equity elements into attitudinal models and providing a deeper insight into how brand perceptions are formed and maintained.

### Mediation effect

Our findings reinforce the critical mediating role of ATT in the relationship between various components of CBBE and PI for EVs. Specifically, ATT is found to significantly mediate the effects of BAS, PQ, and BL on PI, aligning with extant literature that identifies these elements as central to fostering consumer engagement and commitment to a brand [49]. However, BAW does not exhibit the same mediating influence, a divergence that reflects the evolving consumer behavior in the EV market, where awareness may be widespread but does not necessarily convert to intention without favorable associations, quality perceptions, and loyalty. This indicates that brand managers should prioritize cultivating association, quality, and loyalty over mere awareness of EV brands, as these elements are more instrumental in shaping ATT that leads to purchasing decisions.

This result underscores the nuanced impact of each CBBE dimension, suggesting that EV brands need a multidimensional brand strategy that engages consumers on multiple levels. The findings align with the CBBE model [170], which posits that consumer decision-making relies heavily on perceptions of quality and emotional connections, both of which can influence purchase behaviors directly and through attitudinal pathways. In this sense, our study contributes to a deeper understanding of how CBBE translates into consumer behavior specifically within the EV sector, a high-involvement product category where trust, quality perception, and loyalty can be decisive. Moreover, the lack of a mediating role for BAW calls for further exploration. One interpretation may be that in high-involvement purchases, like EVs, mere BAW does not suffice without deeper relational and quality-oriented aspects [166]. This insight informs EV brand management by underscoring the importance of strengthening emotional bonds and perceived value, particularly in an industry marked by rapid technological evolution and heightened environmental consciousness.

### Moderation effect

The study’s findings on PP moderation provide valuable insights into the complex dynamics between ATT and PI in the context of EVs. Our analysis indicates that a lower PP strengthens the positive impact of ATT on PI, whereas a higher PP dampens this relationship. This result is indicative of price sensitivity among EV consumers, who are more likely to act on favorable ATT when they perceive the price as accessible or reasonable. This is consistent with prior research indicating that pricing perception critically influences PI in high-cost products, especially in markets where consumers may still perceive EVs as premium or luxury items. This moderation finding not only aligns with traditional consumer behavior models [15] but also highlights the importance of price positioning in the EV sector, where the perception of price is a determinant of conversion from positive ATT to purchase behavior. As such, brands may benefit from strategic pricing initiatives—such as subsidies, financing options, or perceived value enhancements—to effectively capitalize on positive consumer ATT.

Additionally, as competitive pressures in the EV market increase, brands should consider segmenting their audience by price sensitivity and tailoring marketing communications to reinforce the value-for-money proposition of their offerings. Interestingly, this study’s PP moderation effect both corroborates and diverges from findings in other high-involvement product categories. This divergence suggests a need for further research exploring the boundary conditions of PP’s moderation effect, examining factors like income level, ATT, and familiarity with EVs.

## Conclusion

### Theoretical and managerial implications

This study advances theoretical understanding of consumer behavior in high-involvement, innovation-driven markets by integrating CBBE with TPB within the context of EV adoption. The findings of this study yield significant theoretical contributions while offering actionable insights for key stakeholders in the EV ecosystem.

#### Theoretical implications.

Theoretically, this study makes three pivotal contributions. First, it reveals the differentiated effects of CBBE components—BAW, BAS, PQ, and BL—on PI. The finding that BAW does not significantly influence PI, while BAS, PQ, and BL do, challenges the conventional assumption that familiarity alone drives consumer decisions. In highly dynamic and competitive markets such as China’s rapidly expanding EV sector—marked by aggressive pricing strategies and frequent brand failures—consumers confront elevated levels of uncertainty and risk perception. Within this context, which necessitates substantial consumer involvement and considerable financial investment, mere brand awareness proves insufficient to drive purchase intentions. Consumers transcend basic brand recognition, engaging in comprehensive cognitive and affective processing wherein they rely predominantly on evaluative assessments (e.g., PQ) and emotional attachments (e.g., BL and positive BAS) rather than simple brand familiarity.

Second, the study validates a hybrid CBBE–TPB framework, demonstrating how brand equity elements interact with TPB constructs to shape consumer behavior. ATT is found to partially mediate the relationship between brand components and purchase intention, indicating that brand perceptions influence behavioral intentions through cognitive-affective pathways. This integration extends TPB by illustrating how marketing stimuli—such as branding efforts—shape internal evaluations, thereby contributing to a more holistic understanding of decision-making in the EV context. By bridging CBBE and TPB, this study provides a robust framework for analyzing consumer behavior in high-involvement, sustainability-oriented markets.

Third, this study advances behavioral theory, particularly TPB, by demonstrating the non-significant direct effect of subjective norms on purchase intentions within the EV adoption context. This finding indicates that while social pressures and perceived expectations from significant others traditionally influence behavioral intentions, their direct impact may be substantially attenuated or mediated in high-stakes, high-uncertainty decisions characterized by rapid technological evolution and market turbulence, exemplified by China’s contemporary EV landscape. In contrast to routine consumer goods where social conformity often serves as a primary motivational force, consumers contemplating substantial EV investments appear to prioritize personal assessments of product quality, performance capabilities, and long-term value propositions over immediate social validation. This challenges foundational TPB assumptions regarding the universal applicability and consistent potency of normative influences, highlighting that the relative importance of various intention antecedents varies considerably across product categories and market contexts. These findings suggest that researchers applying TPB to emerging technology adoption or high-involvement purchase decisions should consider contextual moderators and potentially reconceptualize the model to account for the diminished role of social norms in favor of more individualistic, rational evaluation processes in complex market environments.

#### Managerial implications.

The findings of this study offer targeted and actionable strategies for manufacturers, marketers, policymakers, and consumers, providing a roadmap to accelerate EV adoption through coordinated efforts across the industry value chain.

For manufacturers and brand managers, the results underscore the importance of strengthening BAS by linking EV brands with technological reliability, environmental sustainability, and innovation. Strategic storytelling that highlights technological milestones, customer testimonials, and collaborations with green influencers can amplify these associations. To enhance PQ, automakers should provide transparent and interactive product information, including comparison tools, third-party performance evaluations, and visible warranties. Offering extended service packages, in-app diagnostics, and transparent battery performance data can further reinforce consumer trust. Additionally, cultivating BL through post-purchase engagement—such as loyalty platforms, referral programs, and personalized user interfaces that track maintenance and performance metrics—is essential to deepen the emotional bond with the brand.

For marketing practitioners, the insignificant effect of BAW underscores the contextual salience of brand dimensions, indicating that the hierarchical importance of brand equity components fluctuates according to product complexity, risk perception, and market volatility. This provides a more sophisticated understanding of branding theory’s operation within technology-intensive, high-involvement, and rapidly evolving consumer markets, emphasizing that profound engagement and trust-building, rather than superficial recognition, ultimately catalyze purchase behavior. Concurrently, the attenuated influence of SN necessitates a strategic reorientation of communication approaches toward emphasizing individualistic benefits—including long-term economic advantages, personal environmental stewardship, and enhanced mobility experiences—rather than relying predominantly on social validation mechanisms. Personalized marketing communications tailored to distinct consumer lifestyles, complemented by interactive tools such as environmental impact dashboards and financial savings calculators, can enhance the salience and persuasiveness of these value propositions. Additionally, strategically leveraging digital platforms to amplify authentic user testimonials and curated user-generated content can strengthen credibility and foster emotional resonance, thereby compensating for the diminished role of traditional social influence pathways in high-stakes purchase decisions.

From a policy and infrastructure perspective, increasing PBC is crucial to reducing barriers to EV adoption. Policymakers and infrastructure providers should prioritize the expansion of fast-charging networks with real-time availability updates, promote flexible financial schemes such as green loans and leasing models, and support educational campaigns that demystify EV ownership. These efforts can be supplemented by “EV Starter Kits” that provide tutorials, hotline access, and mobile support services to assist first-time users, particularly in underserved regions.

Finally, for potential consumers and communities, experiential access and peer-led trust mechanisms are critical. Initiatives such as local EV test-drive events, peer-sharing programs, and region-specific EV forums can alleviate perceived risks, particularly for first-time buyers. These platforms not only support knowledge exchange but also enhance trust through shared real-world experiences. Collectively, these strategies translate the study’s theoretical insights into practical interventions, offering a comprehensive roadmap to accelerate EV adoption through coordinated efforts across the industry value chain.

In sum, by integrating CBBE with TPB, this study not only enhances theoretical understanding of consumer behavior in high-involvement, innovation-driven markets but also provides actionable strategies for accelerating EV adoption. The findings challenge conventional assumptions, refine existing frameworks, and offer practical solutions for manufacturers, marketers, policymakers, and consumers. Ultimately, this research contributes to both academic discourse and real-world applications, paving the way for sustainable mobility and a greener future.

## Limitations and directions for future research

Despite its contributions, this study has several limitations that warrant consideration. First, potential sampling bias may limit the generalizability of findings, as the sample may not fully represent the diversity of EV consumers across China. Future research should incorporate broader demographic and regional samples or employ longitudinal methodologies to capture evolving consumer ATT over time. Second, the reliance on self-reported data introduces potential biases such as common method variance and social desirability effects. Future studies could mitigate these concerns by employing multi-method approaches, including implicit ATT measures (e.g., Implicit Association Tests) or behavioral tracking data. Third, while this study focuses on consumers in economically developed regions, cross-cultural and socioeconomic comparisons could provide deeper insights into how regional policies, infrastructure availability, and economic factors influence EV adoption across different consumer segments. Fourth, future research should investigate the underlying mechanisms and contextual determinants explaining the non-significant direct effects of BAW and SN on EV purchase intentions. This could involve examining potential mediating or moderating variables, conducting comparative analyses across diverse market conditions, and employing qualitative methodologies to uncover deeper psychological processes in high-involvement consumer decisions. Addressing these gaps could further refine psychological models of technology adoption and inform more effective strategies for fostering sustainable consumer behavior.
